# Editorial: Synthetic biology for the sustainable production of biochemicals in engineered microbes

**DOI:** 10.3389/fbioe.2022.984875

**Published:** 2022-08-15

**Authors:** Shuobo Shi, Jingyu Wang, Hua Ling

**Affiliations:** ^1^ Beijing Advanced Innovation Center for Soft Matter Science and Engineering, College of Life Science and Technology, Beijing University of Chemical Technology, Beijing, China; ^2^ School of Engineering, Westlake University, Hangzhou, China; ^3^ NUS Synthetic Biology for Clinical and Technological Innovation (SynCTI), National University of Singapore, Singapore, Singapore; ^4^ Synthetic Biology Translational Research Programme, Yong Loo Lin School of Medicine, National University of Singapore, Singapore, Singapore; ^5^ Department of Biochemistry, Yong Loo Lin School of Medicine, National University of Singapore, Singapore, Singapore; ^6^ Wilmar-NUS Corporate Laboratory (WIL@NUS), National University of Singapore, Singapore, Singapore

**Keywords:** synthetic biology, cell factory, biochemical, metabolic engineering, CRISPR-Cas, biosensor, carbon fixation

The rapid increase in greenhouse gas (GHG) emission due to the extensive use of fossil resources has necessitated the production of renewable energies and chemicals to sustain the current economic activities while realigning the carbon balance. In particular, the microbial production of biochemicals has provided an attractive route to alleviate the current energy crisis and global climate change. In the past, engineering strategies such as random mutagenesis and conventional selection have long been used for strain development, which is time consuming and at low efficiency. In a fast and efficient manner, synthetic biology approaches are poised to revolutionize strain development for biochemical production in several aspects, including design of non-natural biosynthetic pathways, modular pathway assembly, dynamic sensing and regulation, compartmentalization, pathway balancing and rewiring, cofactor engineering as well as high-throughput screening of biological analytes. This Research Topic brings a collection of recent advances in the development of microbial cell factories for biochemical production using various synthetic biology approaches. Briefly, a general description and summary of the articles in this topic can be found below.

*Escherichia coli* and *Saccharomyces cerevisiae* are now considered the two most attractive microbial cell factories. Masuo et al. engineered *E. coli* for the *de novo* production of raspberry ketone from a simple carbon source with the strategy of increasing the metabolic precursors tyrosine and *p*-coumaric acid. Qin et al. enabled the production of d-penyllactic acid in engineered *E. coli* via fusion protein engineering for an artificial redox self-equilibrium system. In *S. cerevisiae*, Jiang et al. reported that three new target genes *AtGRP7*, *AtMSBP1*, and *AtCOL4* could improve the functional expression of two cytochrome P450 enzymes, CYP76AD1 and CYP736A167. This finding was used to enhance the production of betaxanthin by 1.36-fold and Z-α-santalol by 1.97-fold. Zou et al. reported the effects of the unfolded protein response (UPR) and the metabolic burden in two host strains of yeast. Their results showed that the host strain *S. cerevisiae* W303-1A had better capacity to produce secretory protein, β-glucosidase, which will greatly facilitate the fermentation process from cheap cellobiose feedstock. Raajaraam and Raman proposed an algorithm to optimize co-production of multiple metabolites. This tool was combined with the use of genome-scale metabolic models of *E. coli* and *S. cerevisiae* and aided the design of metabolic engineering strategies.

Non-conventional microbes show many unique physiological and biochemical characteristics and have been developed as new microbial chassis in the synthetic biology era. Yang et al. expressed *Abies grandis* pinene synthase in the cyanobacterium *Synechococcus* sp. PCC 7002 and the engineered cyanobacteria achieved the production of pinene directly from CO_2_. Chang et al. explored *Corynebacterium glutamicum* as a host for efficient 3-hydroxypropionic acid production from acetate by regulating the malonyl-CoA pathway. Rong et al. constructed an engineered *Yarrowia lipolytica* for itaconic acid production from waste cooking oil by expressing cis-aconitic acid decarboxylase. They then investigated the impact of the acetyl-CoA biosynthesis pathway on itaconic acid production.

In some cases, completion of complex tasks within a single organism is extremely challenging. Zhu et al. built an artificial microbial consortium composed of engineered *E. coli* and *Pseudomonas putida* using a strategy named ‘nutrition supply-detoxification’. This co-culture system maximized the advantages of both strains, enabling efficient accumulation of medium-chain-length polyhydroxyalkanoate from glucose–xylose mixtures.

A complex synthetic cell factory is usually composed of many functional components. Now, identification and characterization of basic or improved genetic elements plays a key role in biotechnological and synthetic biology applications. Tong et al. found a variant of amine dehydrogenase via combinatorial active-site saturation test/iterative saturation mutagenesis, and the variant showed a significant improved activity in *E. coli*, which facilitated the biosynthesis of enantiopure amino alcohols. Zou et al. characterized the activity of a putative UDP-glucose 4-epimerase (UGE) in both *E. coli* and *S. cerevisiae*. This work identified its multiple functions in the production of polysaccharides as well as in the formation of structures and activity of polysaccharides, which provided a guide for future biomanufacturing of polysaccharides. Miyake et al. first constructed and optimized a biosensor for short branched-chain fatty acids (SBCFAs) in *S. cerevisiae*, providing the possibility for high-throughput screening of improved producers of SBCFAs. In parallel, a rapid assembly of genetic elements forms the cornerstone in the implementation of synthetic biology designs. Li et al. developed an effective DNA assembly approach that can be applied for NHEJ-based integration in *Y. lipolytica*. This method was subsequently used to optimize the mevalonate pathway for (-)-α-bisabolol production.

Of equal importance, several experts discussed current challenges and future trends in harnessing synthetic biology for the sustainable production of biochemicals in engineered microbes. Feng et al. reviewed how synthetic biology significantly accelerated the development of microbial cell factories for resveratrol, and systematically summarized recent achievements in resveratrol production. Jiang et al. gave a detailed summary of the 5-aminolevulinic acid synthesis in microbial cells, and proposed perspectives for further improvement. Meanwhile, they reviewed the applications and detection methods of 5-aminolevulinic acid. Yu et al. provided an overview of advances in the development and application of CRISPR-Cas systems in C1 metabolizing microorganisms and envisioned various Cas9 variant proteins or effectors, which can be implemented for more powerful genome engineering to promote carbon-negative microbial manufacturing.

This Research Topic presents fabulous examples for the sustainable and cost-effective production of biochemicals in microbial cell factories. As a revolutionary technology, advancing synthetic biology strategies has greatly assisted the strain creation process, maximizing the metabolic pathway of certain microorganisms. However, more advanced tools, especially for non-conventional microbes are in urgent need and insufficient for the relative applications. Furthermore, the discovery or engineering of more functional elements is expected to play significant roles in design and development of complex natural or non-natural biosystems for chemical production.

